# Improved Resistive Switching with Low-Power Synaptic Behaviors of ZnO/Al_2_O_3_ Bilayer Structure

**DOI:** 10.3390/ma15196663

**Published:** 2022-09-26

**Authors:** Chandreswar Mahata, Jongmin Park, Muhammad Ismail, Dae Hwan Kim, Sungjun Kim

**Affiliations:** 1 Division of Electronics and Electrical Engineering, Dongguk University, Seoul 04620, Korea; 2 School of Electrical Engineering, Kookmin University, Seoul 02707, Korea

**Keywords:** ZnO/Al_2_O_3_ bilayer, resistive switching, low-power, synaptic plasticity, short-term plasticity, long-term potentiation

## Abstract

In this work, the resistive switching behavior of bilayer ZnO/Al_2_O_3_-based resistive-switching random access memory (RRAM) devices is demonstrated. The polycrystalline nature of the ZnO layer confirms the grain boundary, which helps easy oxygen ion diffusion. Multilevel resistance states were modulated under DC bias by varying the current compliance from 0.1 mA to 0.8 mA, the SET operations where the low resistance state of the memristor device was reduced from 25 kΩ to 2.4 kΩ. The presence of Al_2_O_3_ acts as a redox layer and facilitates oxygen vacancy exchange that demonstrates stable gradual conductance change. Stepwise disruption of conductive filaments was monitored depending on the slow DC voltage sweep rate. This is attributed to the atomic scale modulation of oxygen vacancies with four distinct reproducible quantized conductance states, which shows multilevel data storage capability. Moreover, several crucial synaptic properties such as potentiation/depression under identical presynaptic pulses and the spike-rate-dependent plasticity were implemented on ITO/ZnO/Al_2_O_3_/TaN memristor. The postsynaptic current change was monitored defining the long-term potentiation by increasing the presynaptic stimulus frequency from 5 Hz to 100 Hz. Moreover, the repetitive pulse voltage stimulation transformed the short-term plasticity to long-term plasticity during spike-number-dependent plasticity.

## 1. Introduction

Emulating brain-inspired synaptic devices has recently attracted attention as a potential application in neuromorphic computing systems [1,2,3,4]. In this context, resistive random access memories (RRAM) with a simple metal–insulator–metal structure have been studied due to their low power consumption, high speed, and high-density memory storage ability [5,6]. However, stable gradual conductance change due to controlled movement of oxygen vacancies is the challenge for integrating memristor in simulating the synaptic function. ZnO and Al_2_O_3_ have shown excellent electrical and optical properties and have been studied successfully in resistive switching. The crystallinity and oxygen content of reactively sputtered ZnO film was found to be largely controlled by the O_2_/Ar ratio during sputtering. Further, it influences the oxygen vacancy (V_O_) formations during the electroforming of conductive filaments [7]. Jing et al. and Gu et al., have explained the mechanism [8,9]. They have shown that the oxygen vacancy defect states $V_{O}^{0}$ and $V_{O}^{2+}$ are originated by the O-deficient Zn orbitals, and the electron and hole injection under the electric field in the ZnO layer controls the formation and rupture of the oxygen-vacancy-related conductive filaments. So, the diffusion of oxygen vacancies by an external electric field controls the resistive switching in the memristor [8]. In this context, Gul et al. introduced an oxygen deficient (ZnO_1−x_) layer intentionally by controlling the O_2_ flow during sputtering between two ZnO layers [10]. However, the filamentary bipolar switching shows a very low ON/OFF ratio. On the other hand, SET and RESET mechanisms were modulated by the external doping of Li and Cu doped ZnO [11,12] although the RESET switching behaviors observed in these cases were stepwise and mostly abrupt. Cobalt dopants in ZnO demonstrated improved resistive switching with a comparatively higher memory window and stable endurance properties, where Co-dopants suppressed uncontrolled oxygen vacancy formations [13]. Recently it was demonstrated that Al_2_O_3_ is a stable electrical insulator with a large bandgap and low leakage characteristics, which benefits from reliable and reproducible switching characteristics [14,15,16,17]. The Al_2_O_3_ interfacial layer plays a significant role in controlling multilevel storage and oxygen vacancies at the oxide/oxide interface [18]. The interlayer formation between the Al_2_O_3_/oxide interface plays an important role, and the migration of oxygen ions controls the redox reactions by localizing the formation and rupture of the conductive filaments [14]. Previous reports show that buffer Al_2_O_3_ introduction with the ZnO layer has improved unipolar resistive switching with a modified memory window [19]. Moreover, inserting the semiconducting layer with an Al_2_O_3_ resistive switching device can be beneficial for attaining low-voltage bipolar memory properties [20]. According to the previous study, the grain boundary is present in the polycrystalline ZnO layer, where loss of oxygen vacancy can be reduced, and a stable switching performance can be achieved by introducing a thin Al_2_O_3_ layer [21]. Wang et al. described the Al_2_O_3_/ZnO bilayer RRAM structure using the ALD technique where both SET and RESET processes were found to be abrupt [22]. On the other hand, Lekshmi et al. used ALD deposited Al_2_O_3_/ZnO bilayer memristor with large variability in cycle-to-cycle switching with ON/OFF ratio less than 10 [23]. Tri-layer Al_2_O_3_/ZnO/Al_2_O_3_ has also been used as a switching layer with uni-polar switching using non-reactive Pt electrodes [19]. Tri-layer memristor was also reported by Kumar et al. where the endurance of bi-polar resistive witching was improved [21]. In earlier reports on Al_2_O_3_/ZnO bilayer and Al_2_O_3_/ZnO/Al_2_O_3_ tri-layer based memristors, the synaptic behavior was not studied in detail, an essential characteristic of neuroplasticity.

A detailed study on gradual conductance change under DC and pulse conditions for short-term and long-term memory behaviors is critically needed for future synaptic device applications. So, this work is focused on the various essential synaptic properties inserting an Al_2_O_3_ interfacial layer between ZnO and the TaN electrode in ITO/ZnO/Al_2_O_3_/TaN. The ZnO layer was deposited by RF sputter using the pure Zn target and O_2_+Ar gas flow. The sputtered ZnO/Al_2_O_3_ interface controls the gradual resistive switching in the memristor. Stable resistive switching and synaptic properties show that gradual conductance change can be achieved by applying electrical stimuli. The memristor can be successfully mimicked as a biological synapse with basic function short-term plasticity (STP), long-term plasticity (LTP), spike rate dependent plasticity (SRDP), spike number dependent plasticity (SNDP), and paired-pulse facilitation (PPF).

## 2. Materials and Methods

Bottom electrode TaN was prepared by sputtering on Si/SiO_2_ substrate with a thickness ~100 nm using the pure TaN target with a Ar flow of 20 sccm. Surface contamination was removed by sequential ultrasonic cleaning with acetone, isopropyl alcohol, and DI water. After cleaning of the TaN surface, the first switching layer of ~3 nm Al_2_O_3_ was deposited using an atomic layer deposition (ALD) system with Al metal precursors as trimethylaluminum (TMA) and H_2_O as ALD oxidant at a substrate temperature of 280 °C. To achieve the target thickness, the recipe of Al_2_O_3_ deposition (total 26 cycles) for 1 cycle was kept as: TMA 0.2 s/Purge 15 s/H_2_O 0.2 s/Purge 15 s. After that, the ZnO layer (~30 nm) was deposited by radio frequency (RF) sputtering system at the 10^−3^ Torr working gas pressure. The gas mixture of Ar + O_2_ at a ratio of 8:12 sccm was used with RF power of 80 W. Finally, 100 μm × 100 μm square patterns were formed through a photo-lithography process. Indium tin oxide (ITO) was deposited (Ar, 8 sccm and RF 60 W) from an ITO target (90:10 wt %) followed by the lift-off process to fabricate the ITO/ZnO/Al_2_O_3_/TaN memristor. The microstructure of the ITO/ZnO/Al_2_O_3_/TaN device was analyzed by the high-resolution transmission electron microscopy (JEOL JEM-F200) along with energy-dispersive x-ray spectroscopy (EDS). Using the Keithley 4200 SCS semiconductor parameter analyzer and 4225-PMU pulse module, DC resistive switching performances and presynaptic pulse-induced synaptic characteristics were evaluated.

## 3. Results and Discussion

### 3.1. Structural Characterization

**Figure 1:**
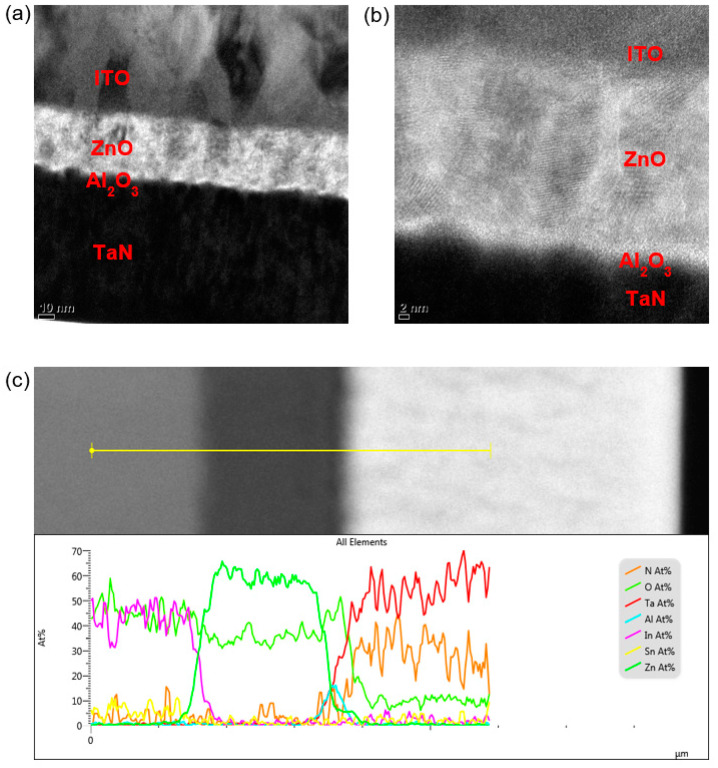
(**a**,**b**) HRTEM image of pristine ITO/ZnO/Al_2_O_3_/TaN resistive switching device; (**c**) EDS line profile of atomic % of N, O, Ta, Al, In, Sn, and Zn scanned from top to bottom electrodes.

The structural properties and thickness of each layer of fabricated ITO/ZnO/Al_2_O_3_/TaN memristor were analyzed by high-resolution transmission electron microscopy (HRTEM) and energy-dispersive X-ray spectroscopy (EDS) line profile. HRTEM shows the switching layer consists of a polycrystalline 30 nm ZnO, and an amorphous 3 nm Al_2_O_3_ bilayer, as shown in Figure 1a,b. A magnified TEM image shows a polycrystalline nature of the ZnO layer which is suitable for electroforming conductive filaments, whereas the amorphous Al_2_O_3_ helps resistive switching characteristics. The EDS line scan atomic % of N, O, Ta, Al, In, Sn, and Zn elements was present in the structure which further confirms the presence of each layer in the fabricated memristor as shown in Figure 1c.

### 3.2. Electroforming and Bipolar Resistive Switching of ITO/ZnO/Al_2_O_3_/TaN Device

**Figure 2:**
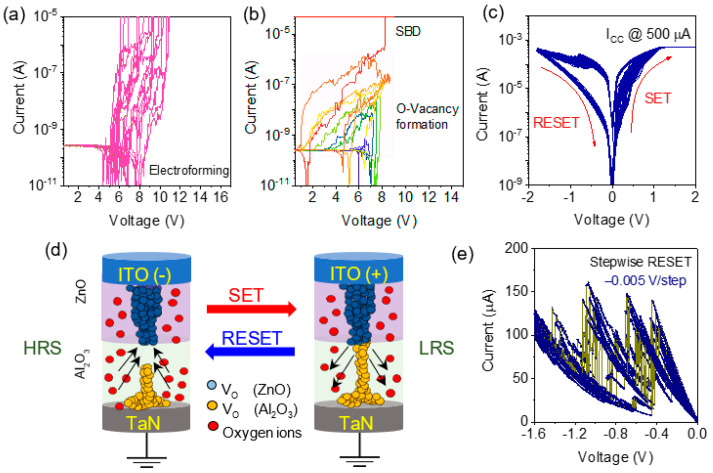
(**a**) Electroforming behavior of ITO/ZnO/Al_2_O_3_/TaN memristor with current compliance of 10 µA; (**b**) charge trapping and O-vacancy formation at increasing ramp voltage stress; (**c**) bipolar resistive switching properties of the memristor at the current compliance of 10 µA; (**d**) schematic illustration of formation and rupture of CFs during the SET and RESET process at the ZnO/Al_2_O_3_ interface; (**e**) 50 cycles of multiple RESET characteristics at a very low sweep rate of −0.005 V/0.5 s show the quantized conductance states.

Bipolar resistive switching was achieved in the ITO/ZnO/Al_2_O_3_/TaN memristor after the electroforming of conductive filaments (CFs) due to the creation of oxygen vacancies under external electric field. In this experiment, electroforming of several memristors were monitored at the current compliance of 10 µA as shown in the Figure 2a. Lower current compliance was selected for protecting the memristor from strong filament formation that cause hard breakdown of the switching layer. The variation of the electroforming voltage may be due to the large area of the device, which forms a different number of CFs under the electric field. The generation of oxygen ions and oxygen vacancies (V_O_) during the external field was further examined by the ramp voltage stress (RVS), where the maximum sweep voltage was increased slowly stepwise by +0.3 V as shown in Figure 2b [24]. Figure 2b shows that the positive charge build-up occurs due to V_O_^+^ formation during RVS and an eventual SBD due to the CFs connecting the top and bottom electrodes. The initial jump in I–V characteristics during RVS at the voltage range of +6.5 to +7.8 V may be due to the presence of polycrystalline ZnO where the strong filament forms easily due to the grain boundary, which provides oxygen vacancies diffusion much easier [25,26]. At higher RVS, the oxygen vacancy creation was slower, possibly due to the Al_2_O_3_ layer and a final soft breakdown, as shown in Figure 2b. Figure 2c shows 200 DC cycles of bipolar resistive switching properties for ITO/ZnO/Al_2_O_3_/TaN memristor, which shows stable endurance properties, and cycle-to-cycle I_ON_/I_OFF_ ratio variations are pretty small. The presence of crystalline ZnO in the switching layer forms a strong filament during electroforming due to the easy movements of oxygen ions [21]. According to Kumar et al., oxygen vacancy in ZnO layers is four times higher than Al_2_O_3_ layers, which play a key role in stabilizing the CFs size and shape [21]. So, when positive voltage is applied to the top electrode during electroforming, thicker cylindrical CFs form inside the ZnO layer. Close to the bottom electrode, Al_2_O_3_ contains thin filaments. During the RESET process at the negative top electrode bias, oxygen ions drift from the TaN electrode (due to the presence of the TaO_x_N_y_ interlayer) easily. The oxygen ions recombine with oxygen vacancy inside the Al_2_O_3_ layer and rupture the filaments (RESET) due to the presence of the weakest part located at the interface of the ZnO/Al_2_O_3_. So, the thin layer of Al_2_O_3_ can stabilize the filament formation and rupture during resistive switching. In the cyclic steps during SET operation, the oxygen ions are again depleted (O-vacancy creation) due to the application of positive bias at the top electrode. The schematic illustration of the SET and RESET operation of the bipolar resistive switching is shown in Figure 2d. Stable DC switching properties of multiples devices are presented in Figure S1 (supplementary materials) which confirm the device-to-device stable variability. During the RESET operation, multiple memory states are achieved at a very slow rate of sweep voltage at −0.005 V/0.5 s as shown in Figure 2e. The stepwise current change is analogous due to the quantum conductance change reported earlier [27,28,29]. So, during the RESET process (25 cycles), the currents reduce from la ow resistance state (LRS) to a high resistance state (HRS) due to the progressive reduction in CFs size behaving as quantum conductance behavior. As shown in Figure 2d, the clear four conductance states indicate multiple stable quantum states suitable for synaptic applications described later in the section below.

**Figure 3:**
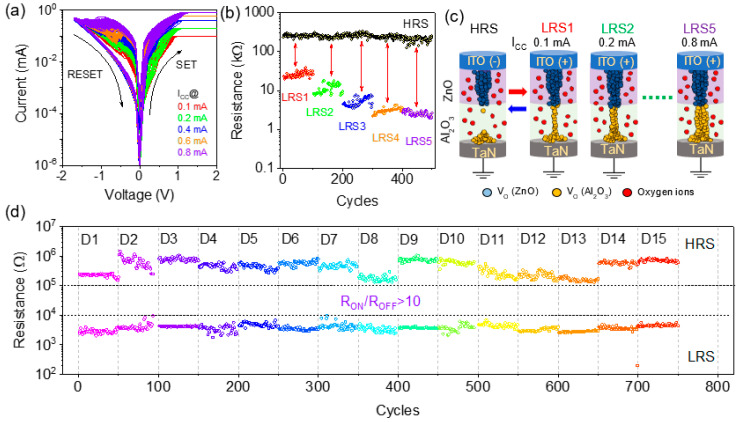
(**a**) Resistive switching behavior of ITO/ZnO/Al_2_O_3_/TaN memristor with varied current compliance at the SET process; (**b**) distribution of HRS and multiple LRS with 100 cycles endurance each; (**c**) filament growth model for oxygen vacancy formation inside ZnO/Al_2_O_3_ switching layer and increasing CFs diameter during multiple LRS; (**d**) endurance characteristics of 15 individual memristor shows an I_ON_/I_OFF_ ratio of >10.

In the ITO/ZnO/Al_2_O_3_/TaN memristor, multiple resistance states were achieved by controlling the current compliance (I_CC_) during the SET process and at a RESET voltage of −1.7 V as shown in Figure 3a,b. Varying the current compliance from 0.1 mA to 0.8 mA, the LRS was changed from 25 kΩ to 2.4 kΩ as depicted in Figure 3b. Resistance states were stable up to 100 DC cycles, indicating the multilevel memory capability of ITO/ZnO/Al_2_O_3_/TaN memristor. The mechanism of multilevel states achieved due to increasing I_CC_ was attributed to the enhancement of the CFs diameter, which caused multiple LRS [3,30]. Figure 3c illustrates filament size modifications under HRS and multiple resistance at LRS1 to LRS 5. So, the control of oxygen vacancy creation inside the Al_2_O_3_ layer during resistive switching by modifying the DC electrical parameter confirms the feasible application of ITO/ZnO/Al_2_O_3_/TaN memristor for the synaptic devices. Device-to-device endurance properties are plotted in Figure 3d, confirming that the R_ON_/R_OFF_ ratio is always >10, suitable for memristor applications.

### 3.3. Synaptic Characteristics under Identical Pulse Sequence

**Figure 4:**
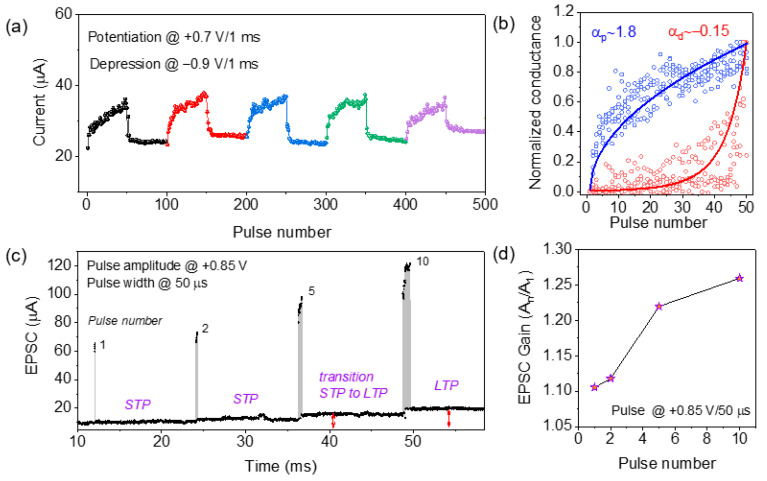
(**a**) Five cycles of potentiation/depression were obtained with 50 identical pulse trains of +0.7 V/1 ms and −0.9 V/1 ms correspondingly; (**b**) linearity factors of potentiation/depression were calculated using Equation (1); (**c**) pulse-number-dependent STP to LTP transition with a pulse amplitude of +0.85 V/1 ms; (**d**) EPSC gain from the ratio of A_n_/A_1_ is plotted.

Furthermore, synaptic properties were emulated for ITO/ZnO/Al_2_O_3_/TaN memristor with identical pulse sequences. Gradual conductance modulation under electrical pulses is an essential synaptic property for neuromorphic applications. A continuous tunable synaptic weight change was observed during potentiation and depression with an identical pulse amplitude of +0.7 V/1 ms and −0.9 V/1 ms correspondingly, as shown in Figure 4a. A fast transition at the first pulse was observed, followed by a gradual change in synaptic weight (current) in both cases similar to the previous reports [31,32]. Although in case of the depression process the rupture of CFs is faster than formation, five consecutive potentiation/depression cycles indicated the stable and reproducible synaptic properties of the ITO/ZnO/Al_2_O_3_/TaN memristor. A normalized conductance vs. pulse number is plotted in Figure 4b for the bilayer device. From multiple cycles, the synaptic weight change (Δ*w*) was fitted, and the nonlinearity factors were obtained by the Equation [32]:

(1) $$G={\left((G^{\alpha }{}_{LRS\;}-G^{\alpha }{}_{HRS\;})\times w+G^{\alpha }{}_{HRS\;}\right)}^{1/\alpha }$$

where *G_HRS_* and *G_LRS_* are the conductance at *HRS* and *LRS*, *w* is varied from 0 to 1, and α is the nonlinearity factor for potentiation and depression. Nonlinearity at potentiation and depression was found to be α_p_~1.8 and α_d_~−0.15, respectively. The transition from synaptic short-term plasticity (STP) to long-term plasticity (LTP) was further monitored by varying the spike number from 1 to 10 with a pulse amplitude of +0.85 V/50 µs as shown in Figure 4c. As shown here, after two spikes, the base voltage (+0.2 V) was recovered, suggesting STP behavior. As shown in Figure 4c, after the application of five successive pulses, a transition from STP to LTP was observed due to the generation of continuous neutral $V_{O}^{0}$ [33]. A similar phenomenon was also confirmed by Luo et al. [34]. However, during spike-number-dependent plasticity (SNDP), the effect of repetitive pulse shows that an eventual LTP behavior can be achieved after 10 stimuli [35]. Repeated pulse strengthens the CFs inside the switching layer through rehearsal, which emulates the biological synaptic behavior as mentioned by Kim et al. [1]. The SNDP ratio A_n_/A_1_ due to the change of excitatory postsynaptic current (EPSC) was increased from 1.1 to 1.25 after applying 1 to 10 spikes, as shown in Figure 4d.

### 3.4. Pulse-Frequency-Dependent and Experience-Dependent Synaptic Properties

**Figure 5:**
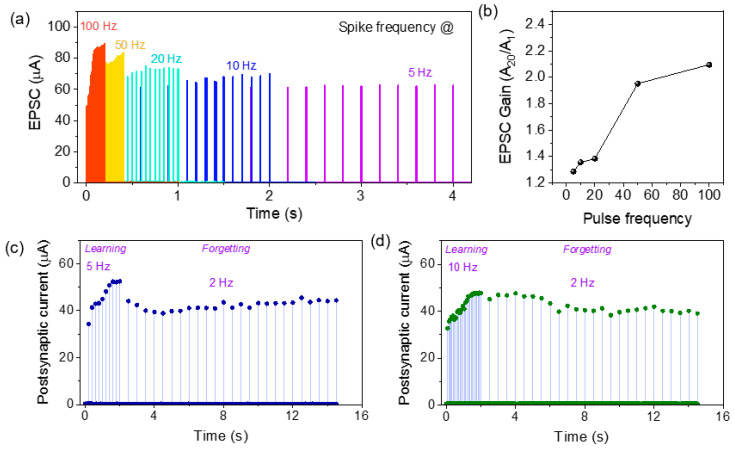
(**a**) Current response of ITO/ZnO/Al_2_O_3_/TaN memristor with consecutive 20 pulse train at the frequency ranges from 5 Hz to 100 Hz with an amplitude of +0.85 V/200 µs; (**b**) frequency-dependent EPSC gain of the memristor; (**c**,**d**) postsynaptic current response shows STP effect at decreasing frequency with the same pulse amplitude of +0.9 V/200 µs.

In neuroscience, spike-rate-dependent plasticity (SRDP) is another essential synaptic function for modifications of memory learning processes that describes the synaptic plasticity dependency on the spike frequency [36,37,38,39]. Figure 5a illustrates the frequency-dependent EPSC response ranges from 5 Hz to 100 Hz with an amplitude of +0.85 V/200 µs in the short-term memory (STM) mode. Each pulse stimulus contains 20 identical pulses with different pulse delay. Current increases significantly at the higher frequencies, whereas the current increment is reduced at the comparatively lower frequency. At the lower frequency of 5 Hz to 10 Hz, the longer recombination time of oxygen vacancies and oxygen ions current is relaxed and tends to reach equilibrium [39]. The EPSC gain is further plotted against the spike frequency in Figure 5b. Synaptic weight change A_20_/A_1_ (A_20_ and A_1_ refer to the current amplitude at the 20th and 1st pulse) clearly depicts that significant potentiation behavior occurred at the higher frequencies. So, the frequency-dependent synaptic weight change in ITO/ZnO/Al_2_O_3_/TaN memristor can mimic the high-pass filter where synaptic functions enhance due to high-frequency input pulse frequency. Experience- or history-dependent plasticity was also emulated on the memristor by applying high- to low-frequency spike trains [36,40]. Series of pulse trains with different pulse frequencies were applied to ITO/ZnO/Al_2_O_3_/TaN memristor with amplitude of +0.9 V/200 µs, and the change in current was observed as shown in Figure 5c,d. The postsynaptic currents increase (learning process) due to the spike train frequency of 5 Hz and 10 Hz at the first stage, whereas at the second stage at spike train frequency of 2 Hz, the current increment or even decreasing trends (forgetting process) were observed. So, the potentiation and depression of synaptic weights are controlled by the pulse train frequency without the application of opposite polarity voltage, which mimics a bio-synapse [37,38]. Similar behavior was reported by Khanas et al. and Yan et al. previously [38,41]. So, ITO/ZnO/Al_2_O_3_/TaN memristor shows the frequency-dependent synaptic weight change, and the potentiation/depression synaptic function can be emulated depending on the history of the frequency.

### 3.5. Paired-Pulse Facilitation(PPF) Emulation

**Figure 6:**
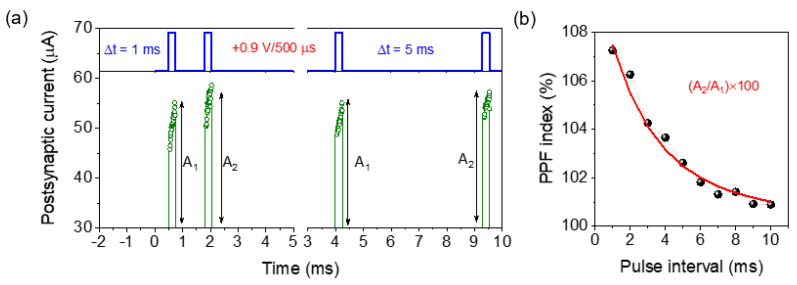
Paired-pulse facilitation short-term memory synaptic function of the ITO/ZnO/Al_2_O_3_/TaN memristor device; (**a**) post-synaptic current response at the pair pulse of +0.9 V/500 µs with a time interval (Δt) of 1 ms and 5 ms; (**b**) experimental PPF index calculated from output current of first and second pulse as (A_2_/A_1_) × 100% along with fitted curve using Equation (2) at the pre-spike time interval of 1 ms to 10 ms.

The short-term synaptic plasticity phenomenon was mimicked for the ITO/ZnO/Al_2_O_3_/TaN memristor with PPF behavior for excitatory response [42,43]. The excitatory postsynaptic current (EPSC) triggered by the second synaptic spike increased compared to the first one during the paired pre-spike pulse signals, as shown in Figure 6a. The EPSC increased sharply due to the presynaptic voltage spike of +0.9 V/500 µs and tended to decay after the first pulse [44]. After applying the successive second pulse, triggered EPSC was found larger than the first one. Depending on the interval time (Δt) of the two pulses, the magnitude of the postsynaptic current at the second pulse increased correspondingly [43]. So, depending on the time interval, the created oxygen vacancies inside the switching layer diffuse back and recombined with oxygen ions. At the shorter interval of 1 ms between two pulses, the recombination process was less compared to the larger pulse interval at 10 ms, and as a consequence, the PPF response increased at the shorter interval. Due to the short interval between the first and second presynaptic spike, the total amount of residual oxygen vacancies increased due to less recombination time. The PPF index A_2_/A_1_ × 100% as a function of Δt is plotted in Figure 6b [42]. As shown in Figure 6b, larger delay time induces a V_o_^+^ and O^−^ recombination, which results in a decreased PPF index. The experimental data was fitted with the decay factor to determine the relaxation time constant by Equation (2):

PPF = C_1_ exp(−Δt/τ_1_) + C_1_ exp(−Δt/τ_2_) (2)

where Δt is the pre-spike interval time, τ_1_ and τ_2_ are the characteristic relaxation time, and C_1_ and C_1_ are the initial facilitation magnitudes [45]. After fitting with experimental data, the value of τ_1_ and τ_2_ was found 6.6 ms and 885 ms, respectively. So, in this study, the gradual change in PPF exponential decay response mimics the biological synaptic properties analogous to the neurotransmitter. Table 1 illustrates the comparison of experimental DC electrical parameters and synaptic properties such as V_SET_, V_RESET_, ON/OFF ratio, and switching type of existing various stacks of ZnO and the Al_2_O_3_-based memristor. It can be seen that ITO/ZnO/Al_2_O_3_/TaN is comparable to the DC characteristics of previously reported memristors. Comparing the postsynaptic current changes in the case of sputtered-ZnO/ALD-Al_2_O_3_ stacks in the present experiment is advantageous regarding various synaptic characteristics.

## 4. Conclusions

The resistive switching characteristics and synaptic properties of the ZnO/Al_2_O_3_ bilayer devices were investigated in detail. The regular grain size of the sputtered deposited ZnO layer and a thin atomic deposited Al_2_O_3_ layer help enhance synaptic properties gradual formation and rupture of the conductive filaments in the ZnO/Al_2_O_3_ interface. Oxygen ions movement due to the presence of the Al_2_O_3_ layer facilitates the controlled formation and recombination of oxygen vacancy near the ZnO/Al_2_O_3_ interface. Multistate memory storage was observed by changing the DC SET current compliance with stable endurance properties. The slow voltage sweep rate at 0.005 V/0.5 s during the RESET process shows the quantized conductance characteristics with stepwise change in current. Achievement of QC-states primarily in the current memristor shows potential applications for high-density storage. Essential synaptic functions, such as potentiation/depression, SRDP, SNDP, and PPF learning rules, are demonstrated on an ITO/ZnO/Al_2_O_3_/TaN device that emulates the biological synapse. Experimental evidence of STP to LTP transition characteristics by frequent presynaptic pulse repetition was observed. Thus, promising electrical properties of the memristor suggest future applications in neuromorphic devices.

## Figures and Tables

**Table 1:** Comparison of resistive switching and synaptic properties of ZnO and the ZnO/Al_2_O_3_-based memristor.

Device Structure	*V*_SET_ (V)	*V*_RESET_ (V)	ON/OFF Ratio	Switching Characteristics	Device-to- Device Reliability	Synaptic Properties	Ref.
Pt/ZnO/Pt	+1.35	−0.95	>10	Abrupt	N/A	N/A	[46]
ITO/ZnO/ITO	+1.6	−2.2	>10	Gradual	N/A	Potentiation/ Depression	[47]
Ag/ZnO/Au	+0.75	−0.75	>10^4^	Abrupt	N/A	N/A	[48]
Al/ZnO/ZnO_1−x_/ZnO/Al	+2.0	−2.5	<10	Gradual	N/A	N/A	[10]
Pt/Al_2_O_3_/ZnO/Al_2_O_3_/Pt	+2.0	−0.75	>10	Abrupt	N/A	N/A	[19]
TiN/Al_2_O_3_/ZnO/Al_2_O_3_/TiN	+1.3	−0.75	>10^2^	Abrupt	N/A	N/A	[21]
TaN/Al_2_O_3_/ZnO/ITO	+1.0	−1.5	>10	Abrupt	N/A	Potentiation/ Depression	[22]
Au/Al_2_O_3_/ZnO/FTO	−1.25	1.5	<10	Gradual	N/A	N/A	[23]
ITO/ZnO/Al_2_O_3_/TaN	+0.75	−1.2	>10	Gradual	Good (Figure 3d)	PD, STP, LTP, SRDP, SNDP, PPF	This work

## Data Availability

Not applicable.

## Supplementary Materials

The following supporting information can be downloaded at: https://www.mdpi.com/article/10.3390/ma15196663/s1, Figure S1: Bipolar resistive switching performances of 16 individual ITO/ZnO/Al_2_O_3_/TaN memristor.
