# Supplementary material for: Improved Resistive Switching with Low-Power Synaptic Behaviors of ZnO/Al2O3 Bilayer Structure
Source: Materials (Basel). 2022 Sep 26;15(19):6663. doi: 10.3390/ma15196663 (PMC9572464; doi:10.3390/ma15196663)
Supplement: Supplementary file 1 [file materials-15-06663-s001.zip › materials-1929357-supplementary.pdf]

Supporting Information

Title: Improved Resistive Switching with Low-Power Synaptic Behaviors of ZnO/Al_2O_3 Bilayer Structure

Authors: Chandreswar Mahata[1], Jongmin Park[1], Muhammad Ismail[1], Dae Hwan Kim[2*], Sungjun Kim[1*]

[1]Division of Electronics and Electrical Engineering, Dongguk University, Seoul 04620, Korea

[2]School of Electrical Engineering, Kookmin University, Seoul 02707, Korea

*****Correspondence: sungjun@dongguk.edu (S.K.), drlife@kookmin.ac.kr (D.H.K.)

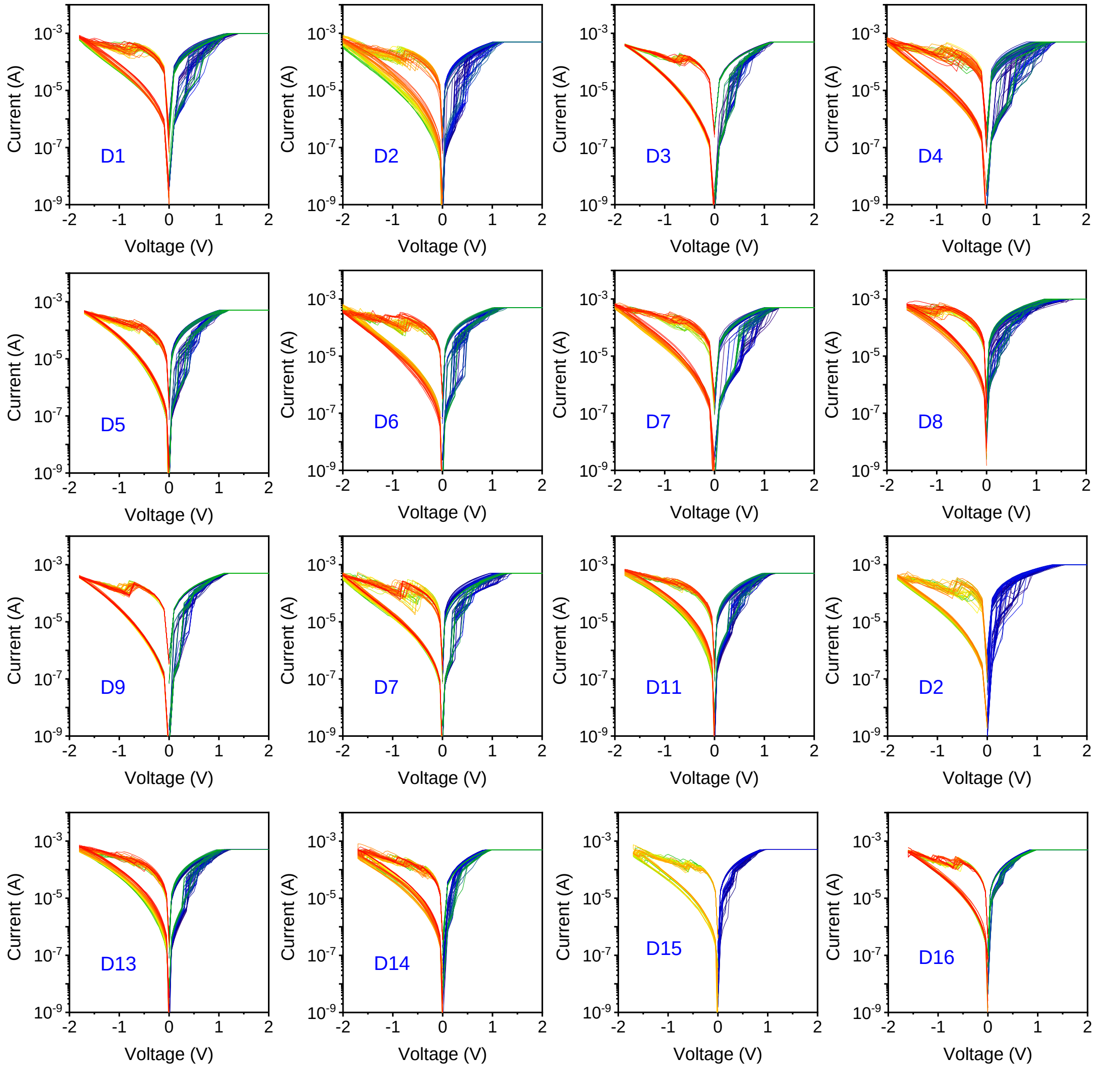

Figure S1: Bipolar resistive switching performances of 16 individual $ITO/ZnO/Al_2O_3/TaN$ memristor.
